# Breaking New Ground towards Innovative Synthesis of Palladacycles: The Electrochemical Synthesis of a Tetranuclear Thiosemicarbazone-[*C*,*N*,*S*] Palladium(II) Complex

**DOI:** 10.3390/molecules29174185

**Published:** 2024-09-04

**Authors:** María L. Durán-Carril, José Ignacio Fidalgo-Brandón, David Lombao-Rodríguez, Paula Munín-Cruz, Francisco Reigosa, José M. Vila

**Affiliations:** Department of Inorganic Chemistry, University of Santiago de Compostela, E-15782 Santiago de Compostela, Spainfrancisco.reigosa@usc.es (F.R.)

**Keywords:** palladium, nickel, Thiosemicarbazones, electrochemical synthesis, crystal structure

## Abstract

The electrochemical oxidation of anodic metals (M = nickel and palladium) in an acetonitrile solution of the thiosemicarbazone ligands (*E*)-2-(1-(4-methoxyphenyl)ethylidene)-N-methylhydrazine-1-carbothioamide (a), (*E*)-2-(1-(p-tolyl)ethylidene)hydrazine-1-carbothioamide (b), and (*E*)-N-phenyl-2-(1-(p-tolyl)ethylidene)hydrazine-1-carbothioamide (c) yielded the homoleptic complexes [ML_2_], **1a**, **1b**, **1c**, and **2c** and [M_4_L_4_], **2a** as air-stable solids. The crystal structures for **1a**, **1b**, **1c**, and **2c** show the ligands in a transoid disposition with the [S,S] and [N,N] donor atom pairs occupying *cis* positions on the nearly square planar coordination plane of the metal. The structure for **2a** of S4 symmetry comprises a tetranuclear palladacycle where the metalated ligands are arranged around a central Pd_4_S_4_ environment: a crown ring with alternating palladium and sulfur atoms. The latter complex is the first example of an electrochemical preparation of a cyclometalated palladium compound, marking a milestone in the chemistry of such species. The compounds have been fully characterized by elemental microanalysis, mass spectrometry, infrared (IR), and ^1^H nuclear magnetic resonance (NMR) spectra.

## 1. Introduction

The palladacycles [1,2,3], a family of cyclometalated compounds for palladium, are highly regarded compounds among organometallic chemists, partly owing to their rich chemistry, but most of all due to their numerous applications related to sensors [4,5], membrane ion transport [6] metallomesogens [7,8], and in catalysis [9,10,11,12,13], where palladium-catalyzed reactions have represented one of the most valuable tools in organic synthesis over the past decades [14,15,16], especially for making carbon–carbon bonds following the Suzuki–Miyaura cross-coupling reaction [17,18,19,20] and as antineoplastic substances [21,22,23,24,25,26,27,28]. Furthermore, quite a few structural aspects are also worth mentioning, such as octonuclear palladacycles [29], related interlocked molecular assemblies [30], and controlled topological transformations of molecular knots [31,32]. Moreover, not only is this aspect of their chemistry interesting, but also the variety of ways of approaching the preparation of the palladium–carbon bond, for which purpose scores of ligands and metal salts have been used; generally, the reaction proceeds in an organic solvent, more often than not with heating under an inert atmosphere. Then, the final mixture needs appropriate work-up to deliver the expected complex.

In the past, we have indulged in the use of several types of solvents and metal salts to obtain the appropriate palladacycles. Also, our research group has widely employed electrochemical synthesis for the preparation of coordination compounds, particularly with ligands bearing the weakly acidic hydroxyl group [33], pyridylmethyl [34], and thiol groups [35], as well as with thiosemicarbazone ligands [36], all of which have been used in conjunction with several types of metal electrodes, plates, and rods of transition (Ni, Cu, Ag, Co, Fe) [37], post-transitional (Zn, Cd, inclusive of Hg, in the liquid state), and non-transition metals (Sn, Pb) [38]; however, the use of palladium metal in the electrochemical synthesis of cyclometalated palladium complexes remains unresolved.

The electrochemical oxidation of metals in the presence of the desired ligand often provides interesting structural variations, since no anions other than the negatively charged ligand and the perchlorate anion from the current carrier are present to complete the linkages to the metal coordination sites [39]. Additionally, unwanted reactions and by-products are avoided by operating with the pure metal and the ligand alone; the applied current suffices to give the appropriate metal oxidation state and to deprotonate the ligand accordingly. In the light of these considerations, we sought to investigate a “cleaner” approach to the synthesis of palladacyles by directly treating the metal itself with the ligand after oxidation by an electrochemical procedure similar to that employed for coordination compounds, breaking new ground in palladacycle chemistry. It is worth noting that in our preparations using standard chemical methods, the presence of ions from metal salts caused synthesis problems in some cases, but in others, it gave rise to completely new and unexpected products, for example, the sandwich coordination of potassium or silver ions, or the hydrolysis of the initial ligand [16,40].

Thus, the purpose was to test the viability of this method, for which we chose a Pd(0) anode immersed in an acetonitrile solution of thiosemicarbazone ligand contained in an electrochemical cell, enabling us to modulate the electrical current conveniently; to the best of our knowledge, there are currently no references to this process having been successful. In any case, and to extend this procedure to other platinum group metals, as well as for comparative purposes, a preparative approach to the analogous nickel complexes was attempted; however, this was to no avail, as we also briefly describe. In accordance with our past results and in order to deepen the capabilities of this synthetic method, we have designed a battery of electrochemical synthesis experiments using palladium foils, as well as nickel foils for comparative purposes, selecting differently substituted thiosemicarbazone ligands that we prepared earlier. This allowed us to first analyze the feasibility of the method, then to determine the adequate conditions, and to come to a conclusion as to whether electrochemical synthesis allows the isolation of species analogous to those obtained by us using the classical chemical method [41]. It would also be interesting to know whether the technique allowed us to distinguish between the formation of the different species, i.e., whether a coordination compound or a palladacycle would be produced. The results obtained allowed us to ensure that the electrochemical method was viable for obtaining palladium(II) complexes and that it allowed us to isolate, in a single step, a species whose crystalline structure proved to be a tetranuclear thiosemicarbazone palladacycle, the first, to our knowledge, using the electrochemical process mentioned above as a method of synthesis.

Thus, the main purpose of this paper was to determine whether a palladacycle could be prepared by an electrochemical procedure, i.e., a direct reaction between the corresponding ligand and the metal, devoid of the conventional metal salts used in this type of syntheses.

## 2. Results

**Scheme 1:**
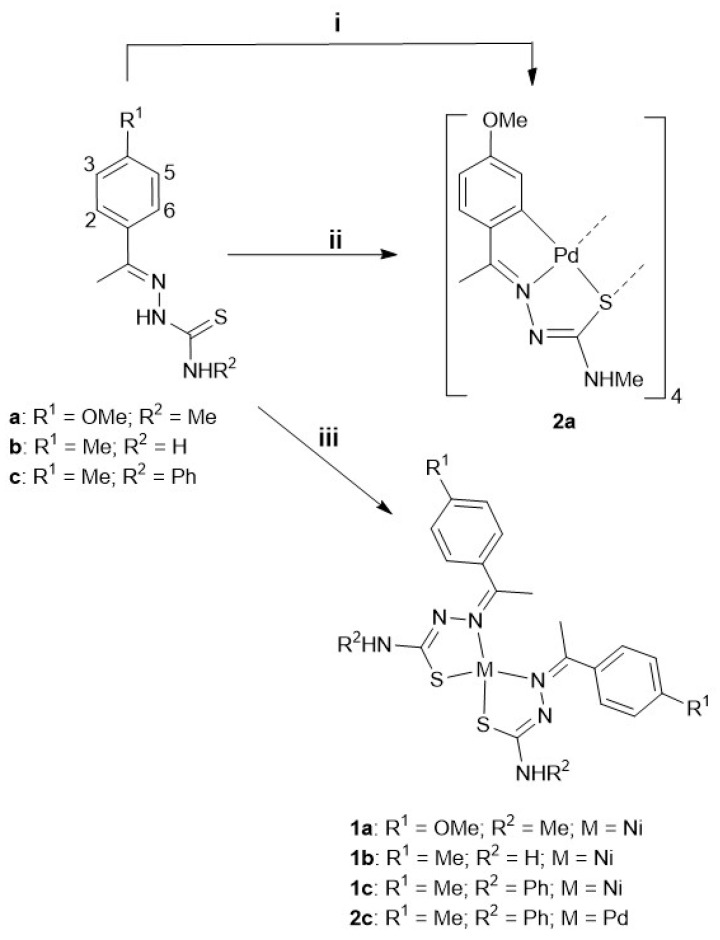
**i**, K_2_[PdCl_4_], EtOH/H_2_O; **ii**, electrochemical cell, M(+), M = Pd/2LH + MeCN/Pt; **iii**, electrochemical cell, M(+), M = Ni/2LH + MeCN/Pt; M(+), M = Pd/2LH + MeCN/Pt.

For the convenience of the reader, the compounds and reactions are shown in Scheme 1. The compounds described in this paper were characterized by elemental analysis (C,H,N,S) and by IR and ^1^H NMR spectroscopy (data in Section 4), as well as by X-ray diffractometric analysis. For comparative reasons, we decided to attempt the preparation of the analogous nickel complexes in the hope of obtaining the cyclometalated nickel compounds, but this did not work; in fact, the results were analogous to those observed in our first attempt to synthesize a palladacycle, compound **2c**. However, after a series of unsuccessful struggles, we succeeded in obtaining the hoped-for palladacycle as a tetranuclear palladium complex, with this being the first case of the synthesis of a palladium metallacycle by an electrochemical procedure. On the other hand, we are aware that this species was already obtained by us, but using a chemical method in accordance with the well-known standard procedures [42]; it goes without saying that what should be emphasized at this early stage is the novelty of the synthesis procedure, not the final product as such, and our efforts will now be directed at extending this groundwork to other cases and varying both the ligand and the metal accordingly.

Thus, the new metal complexes were obtained by the electrochemical oxidation of the appropriate metal (nickel, palladium) in an electrolytic cell containing a solution of the ligand prepared by a condensation reaction of the ketone and thiosemicarbazide; this method represents a simple alternative to other standard chemical procedures.

All complexes obtained are air-stable solids and do not show a tendency to decompose or to oxidize. They are quite soluble in the reaction medium, meaning that in most cases, the resulting solution was concentrated in order to isolate the corresponding complexes. In the cases of complexes **1a**, **1b**, **1c**, and **2c**, elemental analysis shows that the metal ions react with the ligand at a 1:2 molar ratio to afford complexes of the bi-deprotonated ligand (L^2−^).

The electrochemical efficiency E_f_, defined as the amount of metal dissolved per Faraday of charge, was calculated for all the electrochemical processes. In these cases, the values of E_f_ were close to 0.5 mol F^−1^ (see Section 4). These data and the evolution of hydrogen gas from the cathode are consistent with the following reaction scheme:

Cathode:      H_2_L + 2e → H_2_(g) + L^2−^

Anode:      M → M^2+^ + 2e

Overall:      H_2_L + M → [ML] + H_2_(g)

The IR spectra of the ligands showed typical complexes, showing the absence of the hydrazinic ν(N-H)_amide_, ν(N-H)_hydrazinic_, ν(C=N), and ν(C=S) stretch vibrations, ca. 3300, 3170, 1590, and 830 cm^−1^, respectively. Comparison of the spectra of the complexes shows the absence of the ν(N-H)_amide_ and ν(C=S) bands. Both features are in agreement with the deprotonation of the NH group and with the loss of the double-bond character of the C=S group. The ν(C=N) stretch was shifted to a lower frequency with respect to the free ligand consequent on the coordination of the metal to the imine nitrogen atom. 

The main aspects in the ^1^H NMR spectra are the NN*H* resonance and the phenyl resonances that constitute the AA′XX′ spin system. The former signal disappears in the spectra of all the complexes, confirming the deprotonation of the hydrazinic proton. However, in the spectra of complexes **1a**, **1b**, **1c**, and **2c**, the resonances for the AA′XX′ spin system are present, but this is not the case in the spectrum for **2a**. This is consistent with the deprotonation of one of the ortho protons and confirms the metalation of the ligand at the C(2) or C(6) carbon atom.

The fast atom bombardment (FAB) mass spectra of the nickel and palladium compounds showed peaks at *m*/*z* 531 (**1a**), 471 (**1b**), 623 (**1c**), 671 (**2c**), and 1366 (**2a**) for the molecular ions whose isotopic composition suggests complexes of formula [ML_2_] (**1a**, **1b**, **1c**, **2c**) and [M_4_L_4_] (**2a**).

### Molecular and Crystal Structures

**Figure 1:**
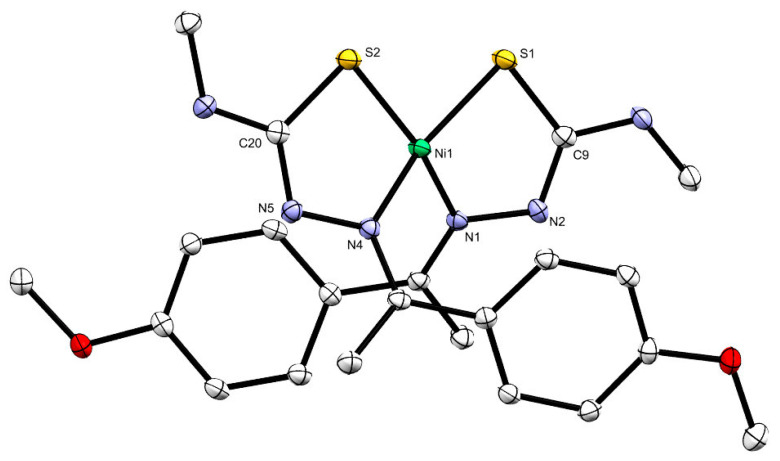
ORTEP drawing of complex **1a** with a thermal ellipsoid plot shown at a 50% probability level. Hydrogen atoms have been omitted for clarity. Selected bond distances (Å) and angles (˚) for 1a are Ni(1)-S(2) 2.1587(6), Ni(1)-S(1) 2.1747(5), Ni(1)-N(1) 1.9156(16), Ni(1)-N(4) 1.9131(16), S(1)-Ni(1)-S(2) 95.52(2), N(1)-Ni(1)-S(2) 162.51(5), N(1)-Ni(1)-S(1) 85.65(5), N(4)-Ni(1)-S(2) 85.78(5), N(4)-Ni(1)-S(1) 163.24(5), and N(4)-Ni(1)-N(1) 98.12(7).

**Figure 2:**
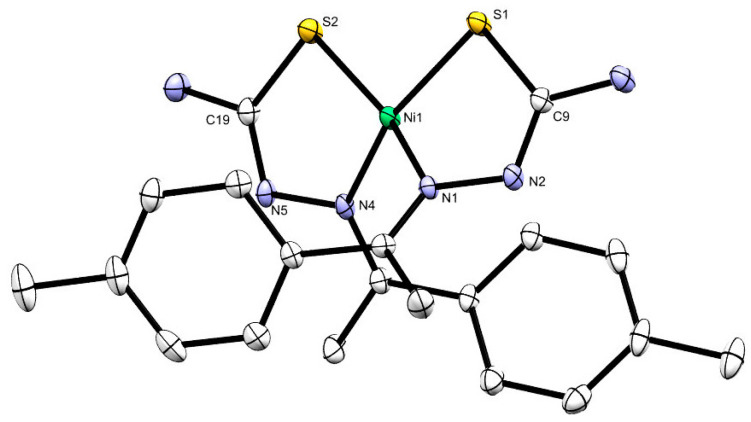
ORTEP drawing of complex **1b** with a thermal ellipsoid plot shown at a 50% probability level. Hydrogen atoms have been omitted for clarity. Selected bond distances (Å) and angles (˚) for 1b are Ni(1)-S(2) 2.1551(6), Ni(1)-S(1) 2.1670(6), Ni(1)-N(4) 1.9233(18), Ni(1)-N(1) 1.9054(19), S(1)-Ni(1)-S(2) 97.27(2), N(4)-Ni(1)-S(2) 86.51(6), N(4)-Ni(1)-S(1) 158.79(6), N(1)-Ni(1)-S(2) 159.65(6), N(1)-Ni(1)-S(1) 85.02(6), and N(1))-Ni(1-N(4) 98.67(8).

**Figure 3:**
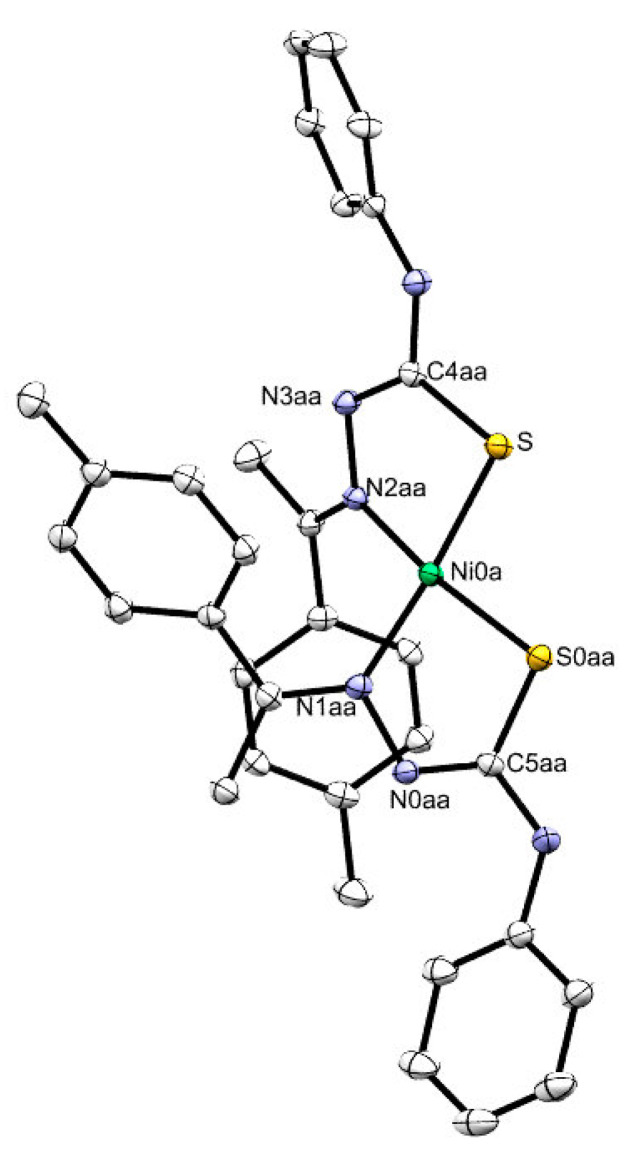
ORTEP drawing of complex **1c** with a thermal ellipsoid plot shown at a 50% probability level. Hydrogen atoms have been omitted for clarity. Selected bond distances (Å) and angles (˚) for 1c are Ni(0a)-S 2.1581(5), Ni(0a)-S(0aa) 2.1567(5), Ni(0a)-N(1aa) 1.9180(14), Ni(0a)-N(2aa) 1.9326(14), S(0aa)-Ni(0a)-S 92.027(18), N(1aa)-Ni(0a)-S 163.19(4), N(1aa)-Ni(0a)-S(0aa) 85.42(4), N(2aa)-Ni(0a)-S 86.13(4), N(2aa)-Ni(0a)-S(0aa) 165.24(4), and N(2aa)-Ni(0a)-N(1aa) 100.44(6).

**Figure 4:**
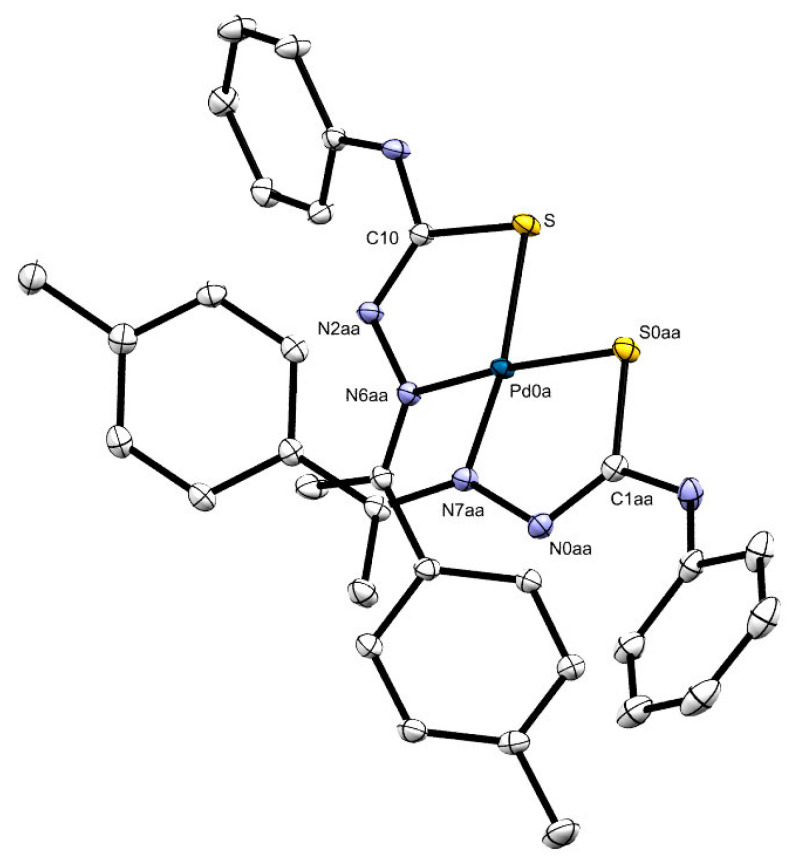
ORTEP drawing of complex **2c** with a thermal ellipsoid plot shown at a 50% probability level. Hydrogen atoms have been omitted for clarity. Selected bond distances (Å) and angles (˚) for 2c are Pd(0a)-S(0aa) 2.2654(3), Pd(0a)-S 2.2652(3), Pd(0a)-N(6aa) 2.0771(11), Pd(0a)-N(7aa) 2.0615(11), S-Pd(0a)-S(0aa) 96.273(12), N(6aa)-Pd(0a)-S(0aa) 169.60(3), N(6aa)-Pd(0a)-S 82.22(3), N(7aa)-Pd(0a)-S(0aa) 81.64(3), N(7aa)-Pd(0a)-S 166.86(3), and N(7aa)-Pd(0a)-N(6aa) 102.13(4).

Crystals were triclinic (**1a**), orthorhombic (**1b**), and monoclinic (**1c**, **2c**), with space groups *P* ®1 (**1a**), *Pbcn* (**1b**), and *P*2_1_/*c* (**1c**, **2c**). The most significant parameters for these compounds are shown in Tables S1–S4, S6–S9 and S11 and Figure 1, Figure 2, Figure 3 and Figure 4. The metal atoms show distorted square planar environments with the ligands in a *cis* configuration; i.e., the phenyl rings point out in close-to-opposite directions. The metal atoms are bonded to two N,S sets from the ligands [43,44]. All bond lengths and angles are within the expected range, with allowance for the *trans* influence of the sulfur atom, which is reflected in the Ni-N distance ca. 2.00 and the Pd-N distance ca. 2.06 Å (cf. the sum of the covalent radii for nickel–nitrogen and palladium–nitrogen, which are 1.86 and 2.01, respectively). The C-S (*ca*. 1.760 Å) and N_amide_-C (1.300 Å) bond lengths show increased single- and double-bond character, respectively, consequent upon the deprotonation of the hydrazinic NH group. The five-membered metal coordination planes (Ni-N-N-C-S) are essentially planar, and the dihedral angle between them is ca. 26.7–13.9°. For example, for compound **1a**, the planes [Ni(1)-N(4)-N(5)-C(20)-S(2)] and [Ni(1)-N(1)-N(2)-C(9)-S(1)] are at an angle of 20.77°.

The angles between adjacent atoms in the coordination sphere of palladium are fairly close to the expected value of 90°, with the smaller values for the bite angles, N-M-S, on each bidentate ligand consequent upon chelation; e.g., for **1a**, N(1)-Ni(1)-S(1) = 85.65(5)˚ and N(4)-Ni(1)-S(2) = 85.65(5)˚. The sum of angles at each metal center, whether nickel or palladium, is ca. 360°.

**Figure 5:**
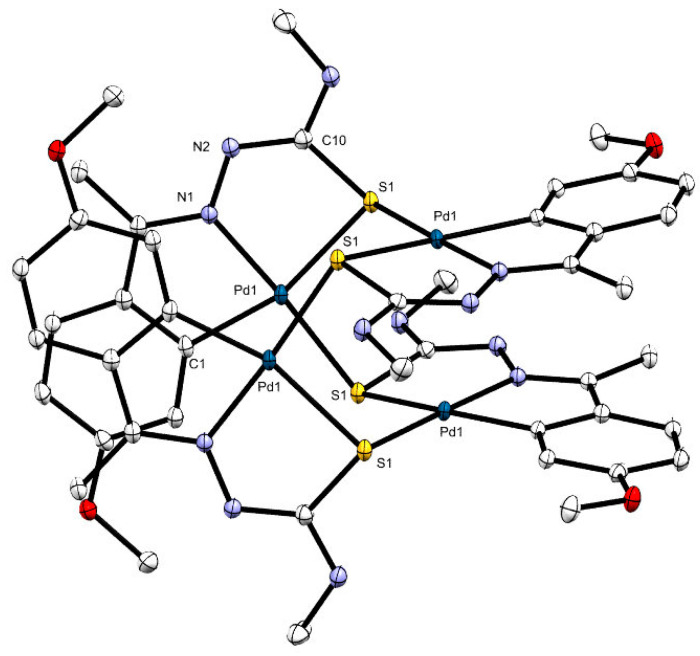
ORTEP drawing of complex **2a** with a thermal ellipsoid plot shown at a 50% probability level. Hydrogen atoms have been omitted for clarity. Selected bond distances (Å) and angles (˚) for 2a are Pd(1)-N(1) 2.0019(14), Pd(1)-C(1) 2.0042(16), Pd(1)-S(1)#1 2.3173(4), Pd(1)-S(1) 2.3674(4), N(1)-Pd(1)-C(1) 81.33(6), N(1)-Pd(1)-S(1)#1 176.57(4), C(1)-Pd(1)-S(1)#1 95.36(5), N(1)-Pd(1)-S(1) 83.16(4), C(1)-Pd(1)-S(1) 162.94(5), and S(1)#1-Pd(1)-S(1) 100.239(10). Symmetry transformations used to generate equivalent atoms: #1 y,-x+1/2,-z+1/2 #2 -y+1/2,x,-z+1/2.

Compound **2a** crystallizes in the tetragonal *P*4_2_/*n* space group; the asymmetric unit comprises a metalated thiosemicarbazone ligand (see Figure 5 below and Tables S5, S10 and S11). The metalated units are located as two perpendicular groups of nearly coplanar antiparallel pairs ca. 3.3 Å apart. As for the structure itself, which has S4 symmetry, the nucleus of the molecule is an eight-membered ring of alternating palladium and sulfur atoms, Pd_4_S_4_; each palladium atom, bonded to a tridentate *C*,*N*,*S* ligand, is coordinated in a close-to-planar geometry to four different atoms: an aryl carbon, an imine nitrogen, a chelating sulfur from the parent ligand, and a bridging sulfur from an adjacent metalated moiety, i.e., [*C*, *N*, *S_chelating_*, *S_bridging_*]. The C(10)–S(1) bond distance, 1.7946(18) Å, is in agreement t with increased single-bond character, and the C(10)–N(2) distance, 1.303(2) Å, with increased double-bond character in the deprotonated form. The Pd–S_bridging_ bond lengths, *trans* to nitrogen 2.3174(4) Å, are shorter than the Pd–S_chelating_ ones, *trans* to carbon 2.3674(4) Å, as a result of the differing *trans* influence of the phenyl carbon and nitrogen atoms of the metalated ligand. 

## 3. Conclusions

In conclusion, we have shown that the field of palladacycle chemistry has not yet reached its outer limits; the well-known technique of electrochemical synthesis, widely employed in the making of coordination compounds, has achieved a new milestone in preparative cyclometalation chemistry, leading to the obtention of a palladacycle, an organometallic compound with metal–carbon bonding. After several attempts to obtain cyclometalated nickel and palladium compounds, which only led to classical metal complexes, it was finally possible to achieve a palladacycle: this is the first example of the synthesis of these compounds by electrochemical synthesis. The crystal structure of **2a** shows unambiguously the certainty of this result. This compound had been previously prepared by our research group, but by a standard chemical method. We can venture that, considering the electrochemical cell parameters were very similar in all cases, it is more than likely that the deprotonation of the ligand at the *ortho* position depends more on the type of metal and the nature of the ligand, e.g., the ring substituents. This issue represents our next challenge, and research in this direction is already underway.

## 4. Experimental Section

### 4.1. General Procedures

Nickel and palladium (Aldrich Chemie, Buchs, Switzerland) were used as plates (ca. 2 × 2 cm). Acetonitrile was purified by standard methods [45]. The preparations were carried out under argon. The ketones and thiosemiacarbazides were used as supplied (all from Aldrich-Chemie, Buchs, Switzerland). Elemental analyses were performed with a Thermo Finnigan elemental analysis, model Flash 1112 (Somerset, NJ, USA). IR spectra were recorded on a Jasco model FT/IR-4600 spectrophotometer (Easton, MD, USA). ^1^H NMR and spectra in solution were recorded in acetone–d_6_ or CDCl_3_ at room temperature on Varian Inova 400 spectrometers (Las Vegas, NV, USA) operating at 400 MHz using 5 mm o.d. tubes; chemical shifts, in ppm, are reported downfield relative to TMS using the solvent signal as reference (acetone–d_6_ δ ^1^H: 2.05 ppm, CDCl_3_ δ ^1^H: 7.26 ppm). Coupling constants are reported in Hz. All chemical shifts are reported downfield from standards. The preparation of the cyclometalated complexes **1a** [10], **1b** [11], **2a**, and **2b** [12] has been reported previously; **2a** and **2b** were used as the perchlorate salts.

Nickel and palladium (Aldrich Chemie) were used as plates (*ca*. 1 × 1 cm).

### 4.2. Preparation of the Ligands

Synthesis of (**a**) 4-Methoxiaceto-phenone (500 mg, 2.11 mmol) and hydrochloric acid (35%, 0.65 cm^3^) were added to a suspension of 4-methyl-3-thiosemicarbazide (222 mg, 2.11 mmol) in water (40 cm^3^) to give a clear solution, which was stirred at room temperature for 4 h. The white solid formed was filtered off, washed with cold water, and dried in vacuo. Yield: 735 mg, 93%. Anal. Found: C: 55.7, H: 6.3, N: 17.6, S: 13.4; C_11_H_15_N_3_OS (237.32 g/mol) requires C: 55.7, H: 6.4, N: 17.7, S: 13.5%. IR(cm^−1^): ν(N-H) 3180, 3363; ν(C=N) 1608; ν(C=S) 834. ^1^H NMR (CDCl_3_): 10.1 (s, 1H, NNH), 8.38 (s, 1H, NHMe), 7.88 (m, 2H, H2/H6, N = 7.9), 6.93 (m, 2H, H3/H5, N = 8.8), 3.79 (s, 3H, OMe), 3.03 (d, 3H, NHMe, ^3^JNHMe = 4.4), 2.25 (s, 3H, MeC=N).

### 4.3. Ligands b and c Were Prepared Analogously

**4-MeC_6_H_4_C(Me)=NN(H)C(=S)NH_2_** (**b**) yield: 641 mg, 83%. Anal. Found: C: 57.8, H: 6.3, N: 20.3, S: 15.5; C_11_H_15_N_3_OS (207.31 g/mol) requires C: 57.9, H: 6.3, N: 20.3, S: 15.5%. IR(cm^−1^): ν(N-H) 3228, 3367, 3153; ν(C=N) 1589; ν(C=S) 816. ^1^H NMR (CDCl_3_): 10.1 *(*s, 1H, NNH), 8.23 (s, 1H, NH), 7.88 (s, 1H, NH), 7.82 (m, 2H, H2/H6, N = 7.9), 7.19 (m, 2H, H3/H5, N = 8.8), 2.32 (s, 3H, C*Me*), 2.27 (s, 3H, *Me*C=N).

**4-MeC_6_H_4_C(Me)=NN(H)C(=S)NHPh** (**c**) yield: 993 mg, 94%. Anal. Found: C: 67.7, H: 5.9, N: 14.8, S: 11.3; C_11_H_15_N_3_OS (283.4 g/mol) requires C: 67.8, H: 6.0, N: 14.8, S: 11.3%. IR(cm^−1^): ν(N-H) 3176, 3241; ν(C=N) 1579; ν(C=S) 812. ^1^H NMR (CDCl_3_): 10.5 (s, 1H, NNH), 7.90 (m, 2H, H2/H6, N = 7.9), 7.57 (m, 2H, H3/H5, N = 8.8), 2.33 (s, 3H, C*Me*), 2.33 (s, 3H, MeC=N).

### 4.4. Electrochemical Synthesis of the Complexes

The nickel and palladium complexes were obtained following the electrochemical procedure described in the literature [33]. The cell consisted of a 100 mL tall-form beaker fitted with a rubber bung, through which the electrochemical leads entered. An acetonitrile solution (40 mL) of the ligand thiosemicarbazone (0.186 mmol), containing a small amount of tetramethylammonium perchlorate as a current carrier (about 10 mg), was electrolyzed (2 h) using a platinum wire as the cathode and a metal 1 × 1 cm (nickel or palladium) plate as the sacrificial anode (caution: although problems were not encountered in this work, all perchlorate compounds are potentially explosive and should be handled in small quantities and with great care). The applied voltages (ca. 20 V) allowed sufficient current flow for smooth dissolution of the metal. The current was maintained at 10 mA. In this case, as we observed in all our experiments in the past, during electrolysis, hydrogen was evolved at the cathode. Under these conditions, the cell can be summarized as M(+), M = Pd/2LH + CH3CN/Pt where LH represents the thiosemicarbazone ligand, with E_f_ ca. 0.50 mol F^−1^. The initial orange solution became darker, until a orange-brownish-colored solution was formed. The reaction mixture was filtered to remove any impurities and allowed to air concentrate at room temperature, resulting in a orange solid that was filtered, washed with acetonitrile and ether, and dried under vacuum.

**[Ni(C_22_H_28_O_2_N_6_S_2_)] 1a**: electrolysis of a solution of the ligand (174 mg, 0.732 mmol) in acetonitrile (40 mL) at 22.4 V and 10 mA for 2 h dissolved 21.5 mg of nickel from the anode, with E_f_ = 0.49 mol F^−1^. At the end of the experiment, the solution obtained was concentrated under vacuum and the resulting solid obtained was isolated, washed with acetonitrile and ether, and dried under vacuum. Yield: 155.7 mg (80%). Anal. Found: C: 49.5, H: 5.3, N: 15.8, S: 13.4; C_22_H_28_N_6_O_2_S_2_Ni (531.32 g/mol) requires C: 49.7, H: 5.3, N: 15.8, S: 12.1%. IR(cm^−1^): ν(N-H) 3349; ν(C=N) 1525. ^1^H NMR (CDCl_3_): 8.65 (s, 1H, NHMe), 7.04 (br, 2H, H2/H6), 6.75 (br, 2H, H3/H5), 3.96 (s, 3H, OMe), 2.54 (s, 3H, NHMe), 1.74 (s, 3H, MeC=N). MS (FAB), *m*/*z*: 531 [M]^+^.

**[Ni(C_20_H_24_O_2_N_6_S_2_)] 1b**: Electrolysis of a solution of the ligand (149 mg, 0.718 mmol) in acetonitrile (40 mL) at 19.1 V and 10 mA for 2 h dissolved 21.0 mg of nickel from the anode, with E_f_ = 0.48 mol F^−1^. At the end of the experiment, the solution obtained was concentrated under vacuum and the resulting solid obtained was isolated, washed with acetonitrile and ether, and dried under vacuum. Yield: 141.6 mg (84%). Anal. Found: C: 49.5, H: 5.3, N: 17.7, S: 13.4; C_20_H_24_N_6_S_2_Ni (471.27 g/mol) requires C: 51.0, H: 5.1, N: 17.8, S: 13.6%. IR(cm^−1^): ν(N-H) 3428, 3351; ν(C=N) 1517. ^1^H NMR (CDCl_3_): 8.56 (s, 1H, NH), 7.82 (s, 1H, NH), 7.32 (m, 2H, H2/H6), 6.39 (m, 2H, H3/H5), 2.37 (s, 3H, CMe), 1.68 (s, 3H, MeC=N). MS (FAB), *m*/*z*: 471 [M]^+^.

**[Ni(C_32_H_32_O_2_N_6_S_2_)] 1c**: Electrolysis of a solution of the ligand (211.4 mg, 0.746 mmol) in acetonitrile (40 mL) at 20.1 V and 10 mA for 2 h dissolved 21.9 mg of nickel from the anode, with E_f_ = 0.51 mol F^−1^. At the end of the experiment, the solution obtained was concentrated under vacuum and the resulting solid obtained was isolated, washed with acetonitrile and ether, and dried under vacuum. Yield: 200.1 mg (86%). Anal. Found: C: 61.5, H: 5.3, N: 13.5, S: 10.2; C_32_H_32_N_6_S_2_Ni (623.46 g/mol) requires C: 61.7, H: 5.2, N: 13.5, S: 10.3%. IR(cm^−1^): ν(N-H) 3342; ν(C=N) 1548. ^1^H NMR (CDCl_3_): 8.65 (s, 1H, NHPh), 7.43 (m, 2H, H2/H6), 7.30 (m, 2H, H3/H5), 7,22 (m, Ph); 6.91 (br, 1H, Ph), 2.33 (s, 3H, CMe), 1.82 (s, 3H, MeC=N). MS (FAB), *m*/*z*: 623 [M]^+^.

**[Pd(C_32_H_32_O_2_N_6_S_2_)] 2c**: Electrolysis of a solution of the ligand (211.2 mg, 0.745 mmol) in acetonitrile (40 mL) at 18.8 V and 10 mA for 2 h dissolved 38.1 mg of palladium from the anode, with E_f_ = 0.48 mol F^−1^. At the end of the experiment, the solution obtained was concentrated under vacuum and the resulting solid obtained was isolated, washed with acetonitrile and ether, and dried under vacuum. Yield: 199.4 mg (83%). Anal. Found: C: 57.2, H: 4.7, N: 12.6, S: 9.5; C_32_H_32_N_6_S_2_Pd (671.19 g/mol) requires C: 57.3, H: 4.8, N: 12.6, S: 9.6%. IR(cm^−1^): ν(N-H) 3327; ν(C=N) 1542. ^1^H NMR (CDCl_3_): 9.33 (s, 1H, NHPh), 7.97 (m, 2H, H2/H6), 7.57 (m, 2H, 2H, H3/H5), 7,22 (m, Ph); 6.94 (t, 1H, Ph), 2.35 (s, 3H, CMe), 1.84 (s, 3H, MeC=N). +MS (FAB), *m*/*z*: 671 [M]^+^.

**[Pd_4_(C_44_H_52_O_4_N_12_S_4_)] 2a**: Electrolysis of a solution of the ligand (90.0 mg, 0.379 mmol) in acetonitrile (40 mL) at 18.8 V and 10 mA for 2 h dissolved 38.9 mg of palladium from the anode, with E_f_ = 0.49 mol F^−1^. At the end of the experiment, the solution obtained was concentrated under vacuum and the resulting solid obtained was isolated, washed with acetonitrile and ether, and dried under vacuum. Yield: 101.2 mg (81%). Anal. Found: C: 38.7, H: 3.7, N: 12.1, S: 9.3; C_44_H_52_N_12_O_4_S_4_Pd_4_ (1366.90 g/mol) requires C: 38.7, H: 3.8, N: 12.3, S: 9.4%. IR(cm^−1^): ν(N-H) 3363; ν(C=N) 1579. ^1^H NMR (CDCl_3_): MS (FAB), *m*/*z*: 1366 [M]^+^.

### 4.5. Crystal Structure Analysis and Details on Data Collection and Refinement

Compound **1a**, CCDC 2362334; compound **1b**, CCDC 2362338; compound **1c**, CCDC 2362339; compound **2c**, CCDC 2362342; compound **2a**, CCDC 2362343 and CCDC 728244 in [42].

## Data Availability

Data are contained within the article and Supplementary Materials.

## Supplementary Materials

The following supporting information can be downloaded at: https://www.mdpi.com/article/10.3390/molecules29174185/s1, Table S1: Bond Lengths for **1a**. Table S2: Bond Lengths for **1b**. Table S3: Bond Lengths s for **1c**. Table S4: Bond Lengths for **2c**. Table S5: Bond Lengths for **2a**. Table S6: Bond Angles for **1a**. Table S7: Bond Angles for **1b**. Table S8: Bond Angles s for **1c**. Table S9: Bond Angles for **2c**. Table S10: Bond Angles for **2a**. Table S11: Crystallographic Data for **1a**, **1b**, **1c**, **2a** and **2c**.
