# Supplementary material for: Breaking New Ground towards Innovative Synthesis of Palladacycles: The Electrochemical Synthesis of a Tetranuclear Thiosemicarbazone-[C,N,S] Palladium(II) Complex
Source: Molecules. 2024 Sep 4;29(17):4185. doi: 10.3390/molecules29174185 (PMC11397458; doi:10.3390/molecules29174185)
Supplement: Supplementary file 1 [file molecules-29-04185-s001.zip › molecules-3101104-supplementary.pdf]

Table S1 Bond Lengths for **1a**

Atom	Atom	Length/Å	Atom	Atom	Length/Å
Ni1	S2	2.1587(6)	C5	C4	1.375(3)
Ni1	S1	2.1747(5)	N5	C20	1.308(2)
Ni1	N1	1.9156(16)	N5	N4	1.401(2)
Ni1	N4	1.9131(16)	N3	C9	1.352(3)
S2	C20	1.755(2)	N3	C10	1.448(3)
S1	C9	1.758(2)	N4	C18	1.299(2)
O2	C14	1.372(2)	C14	C15	1.387(3)
O2	C22	1.427(2)	C16	C15	1.386(3)
O1	C3	1.375(2)	C16	C17	1.393(3)
O1	C11	1.431(2)	C17	C12	1.400(3)
N2	N1	1.402(2)	C17	C18	1.477(3)
N2	C9	1.301(2)	C6	C1	1.395(3)
N1	C7	1.298(2)	C6	C7	1.477(3)
N6	C20	1.350(2)	C1	C2	1.391(3)
N6	C21	1.452(2)	C4	C3	1.387(3)
C13	C14	1.390(3)	C7	C8	1.500(3)
C13	C12	1.380(3)	C3	C2	1.392(3)
C5	C6	1.402(3)	C18	C19	1.493(3)

Table S2 Bond Lengths for **1b**

Atom	Atom	Length/Å	Atom	Atom	Length/Å
Ni1	S2	2.1551(6)	C16	C17	1.475(3)
Ni1	S1	2.1670(6)	C16	C15	1.398(3)
Ni1	N4	1.9233(18)	C16	C11	1.393(3)
Ni1	N1	1.9054(19)	C20	C13	1.505(3)
S2	C19	1.746(2)	C3	C2	1.384(4)
S1	C9	1.760(2)	C3	C10	1.510(3)
N5	N4	1.408(2)	C3	C4	1.388(4)
N5	C19	1.307(3)	C17	C18	1.503(3)
N4	C17	1.303(3)	C15	C14	1.384(3)
N1	N2	1.405(2)	C13	C14	1.391(4)
N1	C7	1.301(3)	C13	C12	1.385(4)
N2	C9	1.313(3)	C6	C7	1.476(3)
N6	C19	1.361(3)	C6	C5	1.394(3)
C1	C6	1.393(3)	C7	C8	1.500(3)
C1	C2	1.391(3)	C5	C4	1.384(4)
N3	C9	1.347(3)	C12	C11	1.389(3)

Table S3 Bond Lengths for **1c**

Ni0a	S	2.1581(5)	C6aa	C4ba	1.477(2)
Ni0a	S0aa	2.1567(5)	C6aa	C6ca	1.400(2)
Ni0a	N1aa	1.9180(14)	C7aa	C9ba	1.393(2)
Ni0a	N2aa	1.9326(14)	C7aa	C8ca	1.508(2)
S	C4aa	1.7515(17)	C8aa	C7ca	1.387(3)
S0aa	C5aa	1.7554(17)	C9aa	C2ca	1.497(2)
N0aa	N1aa	1.4087(19)	C9aa	C5ca	1.479(2)
N0aa	C5aa	1.293(2)	C0ba	C2ba	1.509(3)
N1aa	C4ba	1.302(2)	C1ba	C4ba	1.503(2)
N2aa	N3aa	1.4062(18)	C2ba	C1ca	1.389(3)
N2aa	C9aa	1.298(2)	C2ba	C7ca	1.392(3)
N3aa	C4aa	1.295(2)	C3ba	C3ca	1.379(3)
N	C0aa	1.410(2)	C3ba	C9ca	1.384(3)
N	C4aa	1.368(2)	C5ba	C7ba	1.389(3)
C5aa	N4aa	1.370(2)	C5ba	C9ca	1.402(3)
C0aa	C2aa	1.395(2)	C6ba	C5ca	1.398(2)
C0aa	C1da	1.388(2)	C7ba	C0da	1.394(3)
C1aa	C7aa	1.388(3)	C8ba	C9ba	1.390(2)
C1aa	C6ba	1.385(2)	C8ba	C5ca	1.390(2)
N4aa	C5ba	1.412(2)	C0ca	C4ca	1.387(3)
C3aa	C2aa	1.385(2)	C1ca	C6ca	1.385(3)
C3aa	C0ca	1.384(2)	C3ca	C0da	1.383(3)
C6aa	C8aa	1.400(2)	C4ca	C1da	1.382(3)

Table S4 Bond Lengths for **2c**

Pd0a	S0aa	2.2654(3)	C7aa	C3ba	1.396(2)
Pd0a	S	2.2652(3)	C7aa	C1ca	1.393(2)
Pd0a	N6aa	2.0771(11)	C7aa	C7ca	1.509(2)
Pd0a	N7aa	2.0615(11)	C8aa	C0ba	1.4028(19)
S0aa	C1aa	1.7628(14)	C8aa	C2ba	1.3972(19)
S	C10	1.7594(13)	C8aa	C11	1.4801(19)
N6aa	N2aa	1.4035(15)	C9aa	C1ba	1.3968(19)
N6aa	C	1.2964(17)	C9aa	C	1.4818(18)
N7aa	N0aa	1.4072(15)	C9aa	C5ca	1.3942(19)
N7aa	C11	1.3022(17)	C0ba	C5ba	1.385(2)
N1aa	C10	1.3695(17)	C1ba	C1ca	1.388(2)
N1aa	C3ca	1.4120(17)	C2ba	C8ba	1.392(2)
N2aa	C10	1.2953(17)	C3ba	C5ca	1.3915(19)
N0aa	C1aa	1.2960(18)	C4ba	C6ba	1.3863(19)
C3aa	C5aa	1.394(2)	C	C8ca	1.4984(19)
C3aa	N4aa	1.4112(18)	C5ba	C7ba	1.394(2)
C3aa	C9ba	1.404(2)	C6ba	C3ca	1.3959(18)
C4aa	C4ba	1.387(2)	C7ba	C8ba	1.396(2)
C4aa	C9ca	1.391(2)	C7ba	C6ca	1.509(2)

C5aa	C4ca	1.393(2)	C9ba	C0ca	1.389(2)
C6aa	C3ca	1.3929(19)	C11	C2ca	1.4995(19)
C6aa	C9ca	1.387(2)	C0ca	C0da	1.383(3)
C1aa	N4aa	1.3699(17)	C4ca	C0da	1.385(3)

Table S5 Bond Lengths for **2a**

N1	Pd1	C1	81.33(6)	C5	C6	C1	119.97(16)
S1[1]	Pd1	C1	95.36(5)	C8	C7	C2	123.97(15)
S1	Pd1	C1	162.95(5)	N1	C7	C2	113.10(15)
S1[1]	Pd1	N1	176.57(4)	N1	C7	C8	122.93(16)
S1	Pd1	N1	83.16(4)	C7	N1	Pd1	117.65(12)
C3	C2	C1	119.74(16)	N2	N1	Pd1	123.29(10)
C7	C2	C1	116.69(15)	N2	N1	C7	119.02(14)
C7	C2	C3	123.57(16)	C9	O1	C5	117.79(13)
C2	C1	Pd1	111.05(12)	C10	N2	N1	113.30(14)
C6	C1	Pd1	129.44(12)	C11	N3	C10	122.44(15)
C6	C1	C2	119.40(15)	N3	C10	N2	118.93(16)
C5	C4	C3	119.57(16)	S1	C10	N2	125.98(14)
C4	C3	C2	120.41(16)	S1	C10	N3	114.99(12)
C6	C5	C4	120.85(16)	C10	S1	Pd1[2]	103.04(6)
O1	C5	C4	115.41(15)	C10	S1	Pd1	92.97(6)
O1	C5	C6	123.74(16)				

[1]+Y,1/2-X,1/2-Z; [2]1/2-Y,+X,1/2-Z

Table S6 Bond Angles for **1a**

Atom	Atom	Atom	Angle/°	Atom	Atom	Atom	Angle/°
S1	Ni1	S2	95.52(2)	N3	C9	S1	118.65(15)
N1	Ni1	S2	162.51(5)	N3	C9	N2	117.67(18)
N1	Ni1	S1	85.65(5)	C13	C14	O2	115.76(17)
N4	Ni1	S2	85.78(5)	C15	C14	O2	124.05(18)
N4	Ni1	S1	163.24(5)	C15	C14	C13	120.18(18)
N4	Ni1	N1	98.12(7)	C17	C16	C15	121.60(18)
C20	S2	Ni1	93.70(7)	C16	C15	C14	119.27(18)
C9	S1	Ni1	92.60(7)	C12	C17	C16	117.97(17)
C22	O2	C14	118.15(16)	C18	C17	C16	121.35(17)
C11	O1	C3	117.81(16)	C18	C17	C12	120.67(17)
C9	N2	N1	111.22(16)	C1	C6	C5	117.73(18)
N2	N1	Ni1	117.32(11)	C7	C6	C5	119.50(17)
C7	N1	Ni1	128.55(14)	C7	C6	C1	122.58(17)
C7	N1	N2	114.10(16)	C2	C1	C6	121.72(18)

C21	N6	C20	123.85(17)	C3	C4	C5	120.64(18)
C12	C13	C14	119.96(18)	C6	C7	N1	120.97(17)
C4	C5	C6	120.92(18)	C8	C7	N1	120.42(17)
N4	N5	C20	111.08(15)	C8	C7	C6	118.59(16)
N6	C20	S2	119.06(14)	C4	C3	O1	115.74(17)
N5	C20	S2	123.20(15)	C2	C3	O1	124.46(18)
N5	C20	N6	117.73(17)	C2	C3	C4	119.80(18)
C10	N3	C9	120.00(16)	C17	C12	C13	120.94(18)
N5	N4	Ni1	118.13(12)	C17	C18	N4	118.78(17)
C18	N4	Ni1	126.45(14)	C19	C18	N4	121.75(18)
C18	N4	N5	115.37(16)	C19	C18	C17	119.43(16)
N2	C9	S1	123.68(15)	C3	C2	C1	119.18(18)

Table S7 Bond Angles for **1b**

Atom	Atom	Atom	Angle/°	Atom	Atom	Atom	Angle/°
S1	Ni1	S2	97.27(2)	N3	C9	S1	118.54(17)
N4	Ni1	S2	86.51(6)	N3	C9	N2	118.8(2)
N4	Ni1	S1	158.79(6)	C10	C3	C2	121.7(3)
N1	Ni1	S2	159.65(6)	C4	C3	C2	118.0(2)
N1	Ni1	S1	85.02(6)	C4	C3	C10	120.4(3)
N1	Ni1	N4	98.67(8)	C16	C17	N4	120.4(2)
C19	S2	Ni1	94.67(8)	C18	C17	N4	121.6(2)
C9	S1	Ni1	93.77(8)	C18	C17	C16	117.9(2)
C19	N5	N4	110.86(18)	C14	C15	C16	120.3(2)
N5	N4	Ni1	118.69(14)	C14	C13	C20	121.0(2)
C17	N4	Ni1	126.55(15)	C12	C13	C20	121.1(2)
C17	N4	N5	114.76(18)	C12	C13	C14	117.9(2)
N2	N1	Ni1	118.96(14)	C7	C6	C1	121.4(2)
C7	N1	Ni1	124.72(16)	C5	C6	C1	118.6(2)
C7	N1	N2	116.31(18)	C5	C6	C7	119.9(2)
C9	N2	N1	110.44(18)	C13	C14	C15	121.4(2)
N5	C19	S2	124.39(18)	C6	C7	N1	119.3(2)
N6	C19	S2	117.54(18)	C8	C7	N1	121.8(2)
N6	C19	N5	118.1(2)	C8	C7	C6	119.0(2)
C2	C1	C6	120.0(2)	C3	C2	C1	121.6(2)
C15	C16	C17	120.2(2)	C4	C5	C6	120.5(2)
C11	C16	C17	120.9(2)	C11	C12	C13	121.4(2)
C11	C16	C15	118.5(2)	C5	C4	C3	121.2(2)
N2	C9	S1	122.65(17)	C12	C11	C16	120.4(2)

Table S8 Bond Angles s for **1c**

Atom	Atom	Atom	Angle/°	Atom	Atom	Atom	Angle/°
S0aa	Ni0a	S	92.027(18)	C9ba	C7aa	C1aa	118.34(16)
N1aa	Ni0a	S	163.19(4)	C8ca	C7aa	C1aa	120.74(16)
N1aa	Ni0a	S0aa	85.42(4)	C8ca	C7aa	C9ba	120.92(17)
N2aa	Ni0a	S	86.13(4)	C7ca	C8aa	C6aa	120.54(17)
N2aa	Ni0a	S0aa	165.24(4)	C2ca	C9aa	N2aa	121.83(15)
N2aa	Ni0a	N1aa	100.44(6)	C5ca	C9aa	N2aa	120.45(15)
C4aa	S	Ni0a	94.72(6)	C5ca	C9aa	C2ca	117.58(14)
C5aa	S0aa	Ni0a	94.25(6)	C1ca	C2ba	C0ba	120.46(18)
C5aa	N0aa	N1aa	110.64(14)	C7ca	C2ba	C0ba	121.67(17)
N0aa	N1aa	Ni0a	117.90(10)	C7ca	C2ba	C1ca	117.83(17)
C4ba	N1aa	Ni0a	128.75(12)	C9ca	C3ba	C3ca	120.4(2)
C4ba	N1aa	N0aa	113.26(14)	C6aa	C4ba	N1aa	120.02(15)
N3aa	N2aa	Ni0a	118.57(10)	C1ba	C4ba	N1aa	120.80(16)
C9aa	N2aa	Ni0a	128.17(12)	C1ba	C4ba	C6aa	118.95(15)
C9aa	N2aa	N3aa	113.26(13)	C7ba	C5ba	N4aa	123.90(17)
C4aa	N3aa	N2aa	111.61(13)	C9ca	C5ba	N4aa	116.27(17)
C4aa	N	C0aa	128.66(14)	C9ca	C5ba	C7ba	119.83(18)
N0aa	C5aa	S0aa	123.63(13)	C5ca	C6ba	C1aa	120.63(16)
N4aa	C5aa	S0aa	115.22(13)	C0da	C7ba	C5ba	119.16(19)
N4aa	C5aa	N0aa	121.10(16)	C5ca	C8ba	C9ba	120.40(16)
C2aa	C0aa	N	123.62(15)	C8ba	C9ba	C7aa	121.01(17)
C1da	C0aa	N	116.92(15)	C4ca	C0ca	C3aa	119.30(17)
C1da	C0aa	C2aa	119.34(16)	C6ca	C1ca	C2ba	121.22(17)
C6ba	C1aa	C7aa	120.97(16)	C0da	C3ca	C3ba	119.76(19)
C5ba	N4aa	C5aa	128.22(16)	C1da	C4ca	C0ca	120.35(17)
C0ca	C3aa	C2aa	120.78(16)	C6ba	C5ca	C9aa	120.59(15)
N3aa	C4aa	S	124.06(13)	C8ba	C5ca	C9aa	120.69(15)
N	C4aa	S	115.82(12)	C8ba	C5ca	C6ba	118.55(16)
N	C4aa	N3aa	120.12(15)	C1ca	C6ca	C6aa	121.08(16)
C3aa	C2aa	C0aa	119.76(16)	C2ba	C7ca	C8aa	121.53(17)
C4ba	C6aa	C8aa	121.64(16)	C5ba	C9ca	C3ba	119.9(2)
C6ca	C6aa	C8aa	117.73(16)	C3ca	C0da	C7ba	120.9(2)
C6ca	C6aa	C4ba	120.31(15)	C4ca	C1da	C0aa	120.44(17)

Table S9 Bond Angles for **2c**

Atom	Atom	Atom	Angle/°	Atom	Atom	Atom	Angle/°
S	Pd0a	S0aa	96.273(12)	C5ca	C9aa	C1ba	118.91(12)
N6aa	Pd0a	S0aa	169.60(3)	C5ca	C9aa	C	120.11(12)
N6aa	Pd0a	S	82.22(3)	C5ba	C0ba	C8aa	121.09(13)
N7aa	Pd0a	S0aa	81.64(3)	N1aa	C10	S	114.76(9)
N7aa	Pd0a	S	166.86(3)	N2aa	C10	S	125.62(10)
N7aa	Pd0a	N6aa	102.13(4)	N2aa	C10	N1aa	119.62(12)
C1aa	S0aa	Pd0a	95.05(5)	C1ca	C1ba	C9aa	120.33(13)
C10	S	Pd0a	95.49(4)	C8ba	C2ba	C8aa	120.65(13)
N2aa	N6aa	Pd0a	118.32(8)	C5ca	C3ba	C7aa	121.08(13)
C	N6aa	Pd0a	127.35(9)	C6ba	C4ba	C4aa	121.09(13)
C	N6aa	N2aa	114.32(11)	C9aa	C	N6aa	119.45(12)
N0aa	N7aa	Pd0a	117.84(8)	C8ca	C	N6aa	122.19(12)
C11	N7aa	Pd0a	127.91(9)	C8ca	C	C9aa	118.19(12)
C11	N7aa	N0aa	114.08(11)	C7ba	C5ba	C0ba	121.12(13)
C3ca	N1aa	C10	128.20(11)	C3ca	C6ba	C4ba	119.65(13)
C10	N2aa	N6aa	112.42(11)	C8ba	C7ba	C5ba	117.91(13)
C1aa	N0aa	N7aa	111.28(11)	C6ca	C7ba	C5ba	119.99(14)
N4aa	C3aa	C5aa	124.04(13)	C6ca	C7ba	C8ba	122.04(14)
C9ba	C3aa	C5aa	119.71(14)	C7ba	C8ba	C2ba	121.28(13)
C9ba	C3aa	N4aa	116.25(14)	C1aa	N4aa	C3aa	128.49(13)
C9ca	C4aa	C4ba	119.04(13)	C0ca	C9ba	C3aa	119.82(16)
C4ca	C5aa	C3aa	119.24(15)	C8aa	C11	N7aa	119.58(12)
C9ca	C6aa	C3ca	120.15(14)	C2ca	C11	N7aa	120.92(12)
N0aa	C1aa	S0aa	125.34(11)	C2ca	C11	C8aa	119.27(12)
N4aa	C1aa	S0aa	114.24(10)	C0da	C0ca	C9ba	120.56(16)
N4aa	C1aa	N0aa	120.36(13)	C1ba	C1ca	C7aa	121.16(13)
C1ca	C7aa	C3ba	118.17(13)	C6aa	C3ca	N1aa	116.83(12)
C7ca	C7aa	C3ba	120.70(14)	C6ba	C3ca	N1aa	123.50(12)
C7ca	C7aa	C1ca	121.13(13)	C6ba	C3ca	C6aa	119.54(12)
C2ba	C8aa	C0ba	117.91(13)	C0da	C4ca	C5aa	121.17(17)
C11	C8aa	C0ba	119.87(12)	C3ba	C5ca	C9aa	120.24(13)
C11	C8aa	C2ba	121.94(12)	C6aa	C9ca	C4aa	120.52(14)
C	C9aa	C1ba	120.88(12)	C4ca	C0da	C0ca	119.45(15)

Table S10 Bond Angles for **2a**

Atom	Atom	Atom	Angle/°	Atom	Atom	Atom	Angle/°
N1	Pd1	C1	81.33(6)	C5	C6	C1	119.97(16)
S1[1]	Pd1	C1	95.36(5)	C8	C7	C2	123.97(15)
S1	Pd1	C1	162.95(5)	N1	C7	C2	113.10(15)
S1[1]	Pd1	N1	176.57(4)	N1	C7	C8	122.93(16)
S1	Pd1	N1	83.16(4)	C7	N1	Pd1	117.65(12)
C3	C2	C1	119.74(16)	N2	N1	Pd1	123.29(10)
C7	C2	C1	116.69(15)	N2	N1	C7	119.02(14)
C7	C2	C3	123.57(16)	C9	O1	C5	117.79(13)
C2	C1	Pd1	111.05(12)	C10	N2	N1	113.30(14)
C6	C1	Pd1	129.44(12)	C11	N3	C10	122.44(15)
C6	C1	C2	119.40(15)	N3	C10	N2	118.93(16)
C5	C4	C3	119.57(16)	S1	C10	N2	125.98(14)
C4	C3	C2	120.41(16)	S1	C10	N3	114.99(12)
C6	C5	C4	120.85(16)	C10	S1	Pd1[2]	103.04(6)
O1	C5	C4	115.41(15)	C10	S1	Pd1	92.97(6)
O1	C5	C6	123.74(16)				

[1]+Y,1/2-X,1/2-Z; [2]1/2-Y,+X,1/2-Z

Table S11 Crystallographic Data for 1a, 1b, 1c, 2a and 2c

Compound	**1a**	**1b**	**1c**	**2c**	**2a**
Empirical formula	$C_{22}H_{28}N_6NiO_2S_2$	$C_{20}H_{24}N_6S_2Ni$	$C_{32}H_{32}N_6NiS_2$	$C_{32}H_{32}N_6PdS_2$	$C_{44}H_{52}N_{12}O_4Pd_4S_4$
Formula weight	531.332	471.279	623.477	671.199	1366.907
Temperature/K	100.0	100.0	100.0	100.0	100.00
Crystal system	triclinic	orthorhombic	monoclinic	monoclinic	tetragonal
Space group	P-1	Pbcn	$P2_1/c$	$P2_1/c$	$P4_2/n$
a/Å	8.4095(5)	15.5599(5)	13.4643(4)	13.5768(5)	13.0587(6)
b/Å	10.9812(6)	15.6418(5)	17.5822(5)	17.6994(6)	13.0587(6)
c/Å	13.9708(8)	18.0049(6)	13.4447(4)	13.5741(5)	14.9376(8)
α/°	71.2380(19)	90	90	90	90
β/°	86.873(2)	90	111.3835(10)	113.1076(13)	90
γ/°	77.6178(19)	90	90	90	90
Volume/$Å^3$	1193.04(12)	4382.1(3)	2963.69(15)	3000.17(19)	2547.3(2)
Z	2	8	4	4	2
$\rho_{calc}g/cm^3$	1.479	1.429	1.397	1.486	1.782
μ/mm^{-1}	1.020	1.095	0.829	0.791	1.608
F(000)	557.5	1973.9	1307.1	1373.7	1353.7
Crystal size/mm^3	0.1 × 0.1 × 0.02	0.5 × 0.05 × 0.04	0.1 × 0.08 × 0.07	0.18 × 0.14 × 0.07	0.13 × 0.12 × 0.02
Radiation	Mo Kα (λ = 0.71073)	Mo Kα (λ = 0.71073)	Mo Kα (λ = 0.71073)	Mo Kα (λ = 0.71073)	Mo Kα (λ = 0.71073)
$2\theta/°$	4.96 to 52.74	4.34 to 52.74	4 to 56.56	4 to 61.02	5.18 to 56.7
Index ranges	-10 ≤ h ≤ 10, -13 ≤ k ≤ 13, -17 ≤ l ≤ 17	-19 ≤ h ≤ 19, -19 ≤ k ≤ 19, -21 ≤ l ≤ 22	-17 ≤ h ≤ 17, -23 ≤ k ≤ 23, -17 ≤ l ≤ 17	-19 ≤ h ≤ 19, -25 ≤ k ≤ 25, -19 ≤ l ≤ 16	-17 ≤ h ≤ 17, -17 ≤ k ≤ 17, -19 ≤ l ≤ 19
Reflections collected	35252	46000	80463	96853	61858
Independent reflections	4879 [R_{int} = 0.0476, R_{sigma} = 0.0297]	4484 [R_{int} = 0.0630, R_{sigma} = 0.0303]	7349 [R_{int} = 0.0752, R_{sigma} = 0.0336]	9150 [R_{int} = 0.0414, R_{sigma} = 0.0207]	3184 [R_{int} = 0.0409, R_{sigma} = 0.0141]
Data/restraints/parameters	4879/0/304	4484/0/268	7349/0/374	9150/0/374	3184/0/161
Goodness-of-fit on F^2	1.037	1.044	1.045	1.038	1.044
Final R indexes [I>=2σ (I)]	R_1 = 0.0297, wR_2 = 0.0647	R_1 = 0.0329, wR_2 = 0.0781	R_1 = 0.0317, wR_2 = 0.0677	R_1 = 0.0228, wR_2 = 0.0522	R_1 = 0.0180, wR_2 = 0.0388
Final R indexes [all data]	R_1 = 0.0383, wR_2 = 0.0679	R_1 = 0.0457, wR_2 = 0.0853	R_1 = 0.0473, wR_2 = 0.0751	R_1 = 0.0290, wR_2 = 0.0552	R_1 = 0.0233, wR_2 = 0.0410
Largest diff. peak/hole /e $Å^{-3}$	0.40/-0.53	0.58/-0.50	0.47/-0.38	0.57/-0.52	0.47/-0.50
